# Combined transcriptomic and physiological metabolomic analyses elucidate key biological pathways in the response of two sorghum genotypes to salinity stress

**DOI:** 10.3389/fpls.2022.880373

**Published:** 2022-10-13

**Authors:** Fei Zhang, Feng Lu, Yanqiu Wang, Zhipeng Zhang, Jiaxu Wang, Kuangye Zhang, Han Wu, Jianqiu Zou, Youhou Duan, Fulai Ke, Kai Zhu

**Affiliations:** Sorghum Breeding and Cultivation Physiology Laboratory, Sorghum Institute, Liaoning Academy of Agricultural Sciences, Shenyang, China

**Keywords:** sorghum, resistance, salt stress, transcriptomic, metabolomic, salicylic acid

## Abstract

Sorghum is an important food crop with high salt tolerance. Therefore, studying the salt tolerance mechanism of sorghum has great significance for understanding the salt tolerance mechanism of C_4_ plants. In this study, two sorghum species, *LRNK1* (salt-tolerant (ST)) and *LR2381* (salt-sensitive (SS)), were treated with 180 mM NaCl salt solution, and their physiological indicators were measured. Transcriptomic and metabolomic analyses were performed by Illumina sequencing and liquid chromatography-mass spectrometry (LC-MS) technology, respectively. The results demonstrated that the plant height, leaf area, and chlorophyll contents in *LRNK1* were significantly higher than in *LR2381*. Functional analysis of differently expressed genes (DEGs) demonstrated that plant hormone signal transduction (GO:0015473), carbohydrate catabolic processes (GO:0016052), and photosynthesis (GO:0015979) were the main pathways to respond to salt stress in sorghum. The genes of the two varieties showed different expression patterns under salt stress conditions. The metabolomic data revealed different profiles of salicylic acid and betaine between *LRNK1* and *LR2381*, which mediated the salt tolerance of sorghum. In conclusion, *LRNK1* sorghum responds to salt stress *via* a variety of biological processes, including energy reserve, the accumulation of salicylic acid and betaine, and improving the activity of salt stress-related pathways. These discoveries provide new insights into the salt tolerance mechanism of sorghum and will contribute to sorghum breeding.

## Highlight

Transcriptomic and metabolomic data were combined to reveal the changes in genes and metabolites, which provide new insights into the salt tolerance mechanism of sorghum.

## Introduction

Plant growth can be affected by various abiotic stresses such as drought, cold, heat, and high salinity conditions. To maintain adequate proliferation, plants have evolved intricate mechanisms by which they perceive external signals and respond accordingly (Rahman, 2012). A major event in stress response is the perception and transduction of stress signals through signaling components (Kumar et al., 2011), which results in the activation of numerous stress-related genes and metabolites. In *Arabidopsis* and rice plants, networks interacting during stress defenses have recently been elucidated using transcriptomic analysis (Yao et al., 2018; Chandran et al., 2019). Ionic imbalance and osmotic stress are the two major effects challenged by salt stress. These stresses result in membrane damage and enhance lipid peroxidation and the production of reactive oxygen species (ROS) (Kumar et al., 2013). The performance and mechanism of salt tolerance in plants have also been reviewed in many studies (Fahad et al., 2014; Parihar et al., 2014; Dar et al., 2015; Liang et al., 2017). Sorghum is a major food crop for millions of people worldwide and also suffers from salt stress. Food and Agriculture Organization data have demonstrated that sorghum is currently the fifth most important grain crop in the world (Zheng et al., 2011) and plays an important role in the development and evolution of dedicated energy crops. Sorghum has also evolved a series of responses to salt stress. Lacerda reported that sorghum could reduce Na^+^ and Cl^−^ transport from the roots to the shoot and compartmentalize part of it in specific places in the stems and roots in plants treated with salt (Lacerda et al., 2001). Swami revealed changes in the protein complement of sorghum leaves under salt stress (Kumar et al., 2011). In addition, the use of exogenous components can increase the salt tolerance of sorghum, such as exogenous spermine (Chai et al., 2010), salicylic acid (Mahmood et al., 2010), abscisic acid (ABA) (Amzallag et al., 1990), and glycine betaine (Ibrahim, 2004; Agboma et al., 2008). Like other essential crops, salt stress reduces sorghum yield and production. Several articles have reviewed the physiological and biochemical responses of *sorghum* to salt stress (Almodares and Hadi, 2009; Tari et al., 2013; Anami et al., 2015; Yang et al., 2020). When *sorghum* is subjected to salt stress, cells mobilize multiple components in response. Some studies have demonstrated the stress response mechanism of salt stress in *sorghum* at the level of gene expression (Dugas et al., 2011; Fracasso et al., 2016; Yang et al., 2017), although these are insufficient. Organic solutes such as sugars, organic acids, polyols, and many nitrogen-containing compounds such as amino acids, amides, amino acids, ectoine, proteins, and quaternary ammonium compounds have been proven to play important roles in tolerating the toxicity of ions under salt stress (Ashraf and McNeilly, 2004). Meanwhile, genes also actively respond to salt stress signals, and the expression levels of some genes change dramatically in a short time to synthesize the proteins needed for stress response. Some genes are suppressed under salt stress. Biotechnological methods dedicated to planting transcriptomics analysis provide a valuable opportunity to uncover the basis of plant response to salt stress at the molecular level (Daldoul et al., 2014). The continuous advancement of technology will allow us to obtain the transcriptome expression profile in plant cells and use liquid chromatography/mass spectrometry (LC/MS) technology to identify and analyze metabolites and proteins in the plant (Fiehn, 2002). Integrative omics approaches within large-scale experiments, including genomics, transcriptomics, ionomics, proteomics, and metabolomics, can help decipher the interplay of cellular functions at different levels (Biswapriya et al., 2019). Therefore, we can reveal a series of response mechanisms triggered by cells in response to salt stress. Transcriptomics, along with metabolomics, provides a major tool for characterizing postgenomic processes (Morgenthal et al., 2006). In China, many salt-tolerant sorghum varieties have been selected after many years of breeding. *LRNK1* is a sorghum variety with high yield and salt tolerance and is an ideal plant material to study the salt tolerance mechanism of sorghum. The development of liquid chromatography-mass spectrometry (LC-MS) technology has made it possible to research such resistance in plants. With LC-MS, all nonvolatile polar metabolites, such as amino acids, can be assayed without derivatization (D’Amelia et al., 2018). In particular, short analysis times, selectivity, and almost no matrix interferences have become significant advantages for the LC-MS method when determining metabolites (Thiele et al., 2012). Natera revealed the root lipidome profile response to salt stress in barley by LC-MS (Natera et al., 2016). Kechebar found the phenolic composition changed in salt-treated Saharan trees (Karoune et al., 2017). Therefore, it is possible to use LC-MS to study the metabolite profile response to salt stress in sorghum.

In this study, we reveal changes in genes and metabolites in two extreme sorghum materials under salt stress by combining transcriptomic and metabolomic data under four treatments of salt stress and control. This study will provide technical support for the potential mining of sorghum salt tolerance and selecting salt-tolerant varieties.

## Materials and methods

### Plant materials

Two sorghum restorer lines, *LRNK1* (salt-tolerant (ST)) and *LR2381* (salt-sensitive, SS), were used as plant materials. While they had different sensitivities to salt, the crop maturity period, growth rate, and phenotypic traits of the two sorghum genotypes (*Bicolor* L. Moench) were similar. *LRNK1* and *LR2381* seeds were collected from the sorghum seed resource bank in the Liaoning Academy of Agricultural Sciences with ID: 011427 and ID: 010362, respectively. After disinfection (surface-sterilized with 1% sodium hypochlorite for 5 min followed by rinsing three times with distilled water), the seeds were planted in a hydroponic beaker filled with sorghum hydroponic culture (Kimura solution, **Supplementary Table S1**) under artificially simulated climatic conditions: day/night temperatures of 28°C/25°C, approximately 50% relative humidity (Rh), photoperiod of 14:10 h of light (1,500 μmol·m^−2^·s^−1^)/dark in April 2019 in an artificial climate room in Liaoning Provincial Academy of Agricultural Sciences.

### Salt treatment and sample collection

Sorghum plants at the six-leaf stage were used for the salt treatment experiment. For each variety, 144 plants were randomly selected and then divided into six hydroponic beakers with 24 plants in each. These six hydroponic beakers were then randomly divided into two groups: salt treatment (T) and control (C), with three hydroponic beakers in each group. For group T, both LRNK1 and LR2381 were treated with an additional 180 mM NaCl in the basic sorghum hydroponic culture, which was based on previous salt stress simulations at 60, 120, 180, and 240 mM NaCl. The two extremes of 180 mM NaCl showed significant phenotypic differences between the salt stress and control treatments. The salt-sensitive lines survived, while the salt-sensitive lines died under 240 mM NaCl simulations. In addition, the salt stress adversity simulated by 180 mM NaCl most closely matched the actual production environment. For group C, both LRNK1 and LR2381 were treated with basic sorghum hydroponic culture (no NaCl supplement). A total of four sets of samples were prepared: LRNK1-control (STC), LRNK1-salt treatment (STT), LR2381-control (SSC), and LR2381-salt treatment (SST). After 72 h of cultivation, six plant samples were randomly selected from each hydroponic beaker and the fifth leaf at tillering on each plant was sampled (18 leaves obtained in each group). These 18 leaves were pooled and divided into six parts in each group, three of which were used for RNA-seq analysis and three for metabolomic analysis. All sampled leaves were immediately placed in liquid nitrogen for 5 min, stored at −80°C, and tested within 3 to 4 weeks. The root tissue of each group was sampled to determine the physiological indicators.

After the hydroponic salt stress sampling was completed, the seedlings of the four groups of STC, STT, SSC, and SST were transplanted into prepared pots treated with the same salinity as the hydroponics for cultivation until maturity, and the sorghum growth phenotype parameter was recorded.

### Determination of plant physiological indicators

The plant height and leaf area (the fifth leaf at tillering) of seedlings at the six-leaf stage with and without salt treatment were measured according to a previous report (Iqbal et al., 2010). Fresh leaves were determined by an electronic balance to obtain the fresh mass (FM) and dried at 105°C for half an hour and at 70°C for 48 h to achieve constant weight. The dry mass (DM) was recorded. Turgid mass (TM) was determined after the leaves had been immersed in distilled water for 12 h. The FM, DM, and TM of root tissues were also determined. The relative water content (RWC) of a plant tissue is expressed by RWC (%) = [(FM − DM)/(TM − DM)] * 100%. For the leaf chlorophyll content determination, the ethanol-acetone method was used as described by Zhang (1992). The same experimental protocol was repeated three times.

### Total RNA extraction and sequencing

Total RNA was extracted using an RNAprep pure Plant Kit (Tiangen, Beijing, China) according to the manufacturer’s instructions, and the RNA quality was evaluated by gel electrophoresis. Total RNA was reverse transcribed to cDNA by a QuantScript RT Kit (Tiangen, Beijing, China). An efficient VAHTS^®^ mRNA-seq v2 Library Prep Kit for Illumina (Vazyme Biotech, Nanjing, China) was then used to construct the sequence libraries. All 12 mRNA libraries (six groups with three biological replicates in each group) obtained were evaluated by an Agilent 2100 Bioanalyzer (Agilent Technologies, USA) and the qRT-PCR method. Finally, all mRNA-seq libraries were sequenced on an Illumina HiSeq 4000 sequencing platform with pair-end 2 × 150 bp mode to obtain sequencing data.

### mRNA sequence data processing

The sequencing data were filtered using FastQC (version 0.11.5) with default criteria. Low-quality reads and adaptor reads were removed from the raw data, and the clean data were assembled and mapped to the sorghum assembly genome (https://www.ncbi.nlm.nih.gov/genome/term=Sorghum+bicolor) using HISAT2 (version 2.1.0) (Kim et al., 2015). The value of FPKM (expected number of fragments per kb of exon per million of reads) of reads in each sample was calculated using Cufflinks (version 2.2.1) (Roberts et al., 2011). Principal component analysis (PCA) and Pearson’s correlation analysis were performed based on the FPKM of reads. The differentially expressed genes (DEGs) by different comparisons were identified using DESeq2 (version 1.24.0) (Anders, 2010), with the criteria of the corrected *p*-value (padj) <0.05 by the negative binomial distribution test and |log_2_(fold change (FC))|>0. Genes with log_2_FC >0 and log_2_FC <0 were identified as up- and downregulated DEGs, respectively. Hierarchical clustering based on the expression profiles of DEGs was presented by pheatmap (version 1.0.10). DEGs enriched into modules correlated with the phenotypes were separately subjected to the enrichment analysis for Gene Ontology (GO) and Kyoto Encyclopedia of Genes and Genomes (KEGG) pathways (Kanehisa et al., 2007). Significant GO biological processes (BP) and KEGG pathways were identified with the criterion of *p* < 0.05.

### Nontargeted liquid chromatography-mass spectrometry

The chromatographic column (Accucore HILIC column; Thermo Fisher Scientific) was maintained at 40°C with an injection volume of 5 µl. The mobile phase (0.3 ml/min flow rate) consisted of 0.1% formic acid, 95% acetonitrile, and 10 mM ammonium acetate in solvent A, and 0.1% formic acid, 50% acetonitrile, and 10 mM ammonium acetate in solvent B. The gradient elution phase of solvent A was 98% for the first 1 min, decreased to 50% during 1–17 min, stabilized for 0.5 min (17.5 min), returned to 98% after 17.5–18 min, and stabilized at 98% for the last 2 min (18–20 min). A 5-min interval was set for the equilibrating instrument. All samples were analyzed using a Q Exactive (QE) HF-X mass spectrometer detector (Thermo Fisher Scientific) in positive and negative (polarity) ionization modes (spray voltage of 3.2 kV) with MS/MS data-dependent scans (mass range of 100–1500 m/z). The parameters of the electrospray ion source were set as 320°C drying gas temperature, 35 arb sheath gas flow rate, and 10 arb Aux gas flow rate. System stability and accuracy were validated using QC samples with an interval of five samples.

### Nontargeted LC-MS analysis

The data were recorded using the compound discoverer (CD) software, and the parameters, including retention times and mass charge ratio of compounds, were analyzed and corrected. The relative standard deviation (RSD) and signal-to-noise (S/N) ratio were analyzed. After feature screening, candidate features (RSD >30%, S/R ratio <3, signal intensity >10^5^) were retained and aligned against mzCloud (https://www.mzcloud.org/). QC samples were used for data normalization, and the relative expression of the metabolites was obtained and used for further analysis. The resulting three-dimensional data involving the peak number, sample name, and normalized peak area were fed into R package metaX for PCA and projections to latent structures-discriminant analysis (PLS-DA) (Wen et al., 2017). A Student’s *t*-test was used to compare peak areas of three metabolites, with the threshold of variable importance in the projection (VIP) value >1, fold change (FC) ≥2 (|log2FC|≥1), and *p* < 0.05. A hierarchical clustering analysis was then performed to present the expression patterns of the differential metabolites. Subsequently, the correlations between the differential metabolites were calculated by R software (v3.1.3). Differential metabolites with significant correlations (false discovery rate, FDR <0.05) were retained and used for enrichment analysis of the KEGG pathway, with significant criteria of *p*adj < 0.05.

### Soluble sugars, salicylic acid, and betaine

#### Soluble sugars

Soluble sugar in plant leaves was determined with the methods used by Zhang (1992) with minor modifications. Plant leaves (0.25 g) were placed in a boiling water bath for 1 h. Total soluble sugar content was analyzed with the phenol-sulfuric method after hydrolysis of starch using perchloric acid.

#### Salicylic acid content

In total, 1.5 g of seedling leaves was placed in a grinder, and 4 ml of 80% methanol was added to grind them. PVP with 0.2 times the quality of the product was continuously ground into a homogenate. The homogenate was leached in a 4°C refrigerator for 12 h and centrifuged (10,000 r/min, 4°C, 20 min); the supernatant was transferred to a Petri dish and placed in a lighted incubator to dry. A total of 2.5 ml was added to the residue with 80% methanol and leached in a 4°C refrigerator for 12 h, followed by centrifugation. This was repeated three times. After the liquid in the petri dish dried, 2.5 ml of 100% methanol was added to dissolve the dry sample, which was placed into a centrifuge tube and centrifuged (10,000 r/min, 4°C, 10 min). The supernatant was then filtered with a 0.22-μm organic filter membrane and placed in a refrigerator at 4°C. The salicylic acid (SA) content was determined by high-performance liquid chromatography.

#### Betaine

The sample was predried at 105°C to a constant weight, 0.4 g of the dried sample was weighed, 50 ml of glacial acetic acid was added, and heated to dissolve. A total of 25 ml of mercuric acetate solution was added and allowed to cool, then two drops of crystal violet indicator were added and titrated with perchloric acid standard solution (0.1 mol/L) until the solution turned green. The titration result was corrected with a blank test, and high-performance liquid chromatography was used to determine the content of SA.

### Statistical analysis

Differences in expression levels were investigated using a one-way analysis of variance (ANOVA). The means of the values were compared using Tukey’s multiple comparison tests. A *p*-value of <0.05 was considered significant. For conjoint analysis, the correlation analysis between the top 100 DEGs and the top 50 differential metabolites was performed, and the *p*-value was calculated. KEGG enrichment analysis was then performed on these significantly related DEGs and differential metabolites. A *p*-value of <0.05 was considered significant.

## Results

### Effects of salt stress on plant physiological indicators

**Figure 1:**
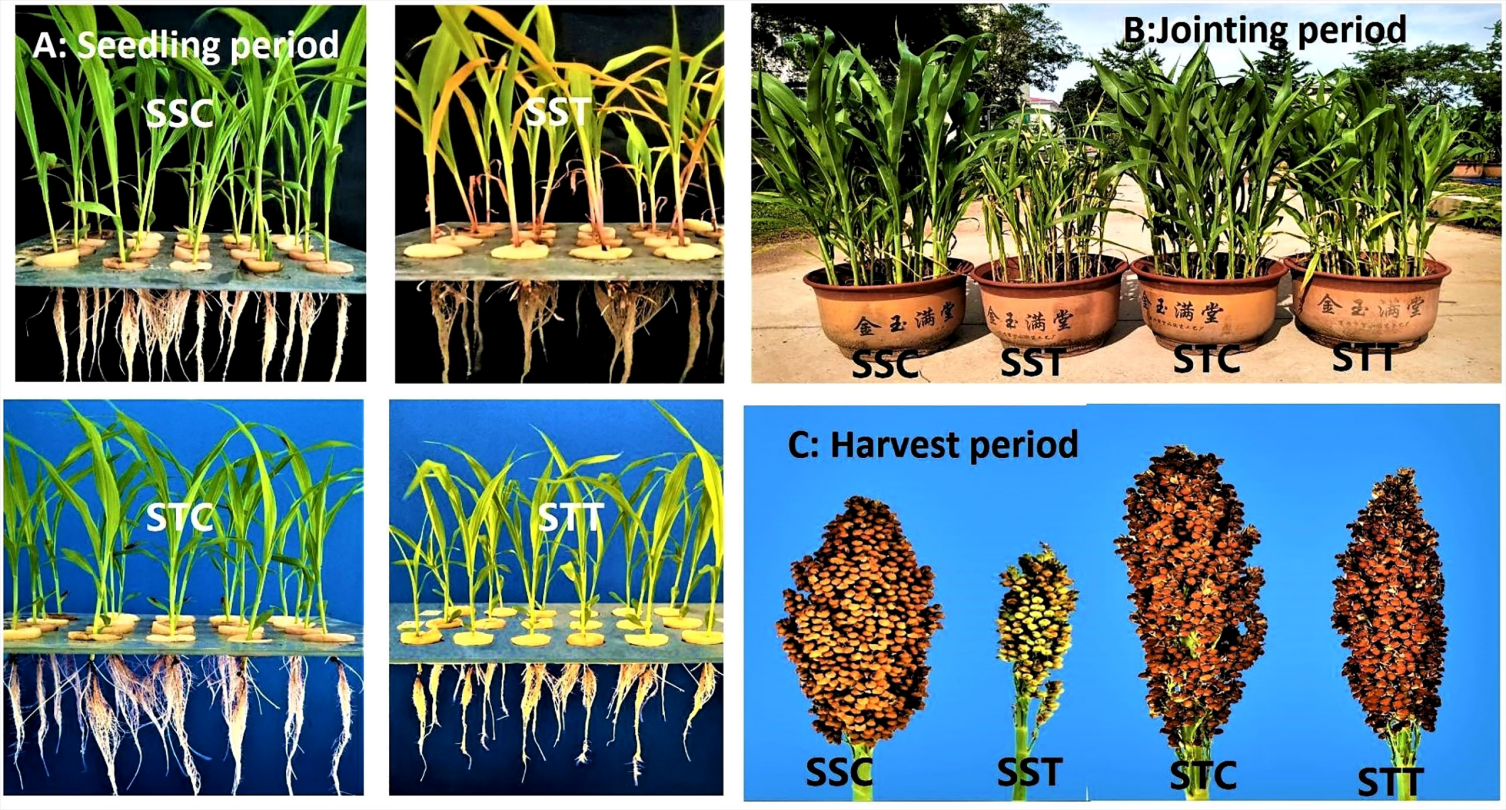
Phenotypic differences between salt-tolerant and salt-sensitive genotypes of sorghum under control and salt stress at seedling period, jointing period, and mature period **(A)** Control vs salt stress phenotypic differences at seedling period. **(B)** Control vs salt stress phenotypic differences at jointing period. **(C)** Control vs salt stress phenotypic differences at harvest period.

Physiological indicators showed that plant height, leaf area, and chlorophyll contents showed no significant difference between the two sorghum varieties under normal conditions (**Figure 1**). However, after salt treatment, these three indicators in the salt-tolerant sorghum *LRNK1* were significantly higher than in the salt-sensitive *LR2381*. In addition, RWC in *LR2381* leaves and roots had significantly lower contents than that in *LRNK1*, which revealed the serious water loss in the salt-sensitive *LR2381* due to salt stress. The morphological differences between salt-tolerant and salt-sensitive sorghum under salt stress are obvious in the seedling period, jointing period, and mature period (**Table 1** **;** **Figure 1**).

**Table 1:** Plant physiological indicators for plant height, leaf area, leaf (FM, DM, TM, RWC), root (FM, DM, TM, RWC), and chlorophyll of salt-sensitivity (SS).

	Plant height	Leaf area	Leaf				Root				Chlorophyll
			FM	DM	TM	RWC	FM	DM	TM	RWC	
SSC	34.65 a	152.23 a	1.13 a	0.13 a	1.90 a	85.3 a	1.13 a	0.14 a	1.20 a	83.6 a	1.54 a
SST	12.86 c	46.05 c	0.02 c	0.02 b	1.71 b	74.2 b	0.15 c	0.02 b	1.01 b	71.2 b	1.12 c
STC	33.57 a	151.24 a	1.02 a	0.13 a	1.87 a	85.6 a	0.94 a	0.12 a	1.19 a	83.1 a	1.53 a
STT	20.43 b	82.79 b	0.18 b	0.07 b	1.85 a	84.7 a	0.43 b	0.06 b	1.15 a	82.7 a	1.45 b

Different lowercase letters (a–c) indicate group differences at the 0.05 level (LSD postanalyses for salt-sensitive (SS) and salt-tolerant (ST) sorghum genotypes under control (C) and salt treatment (T) groups).

### Illumina sequencing data summary

A total of 458.10 Mb of raw reads from the 12 libraries were generated, with an average sequencing error rate of 0.02% and an average GC content of 54.74% (**Supplementary Table S2**). The average data ratio of sequencing quality to Q20 and Q30 was 97.96% and 94.34%, respectively. After read mapping analysis, an average of 92.59% of reads were uniquely mapped to the sorghum genome sequence (**Supplementary Table S2**). PCA results showed that the biological repeat samples in each group are clustered together (**Supplementary Figure S1A**). Sample-to-sample correlation analysis results showed a similar result with PCA, indicating good consistency and reproducibility between biological repetitions (**Supplementary Figure S1B**).

### DEGs between salt treatment and control group

**Figure 2:**
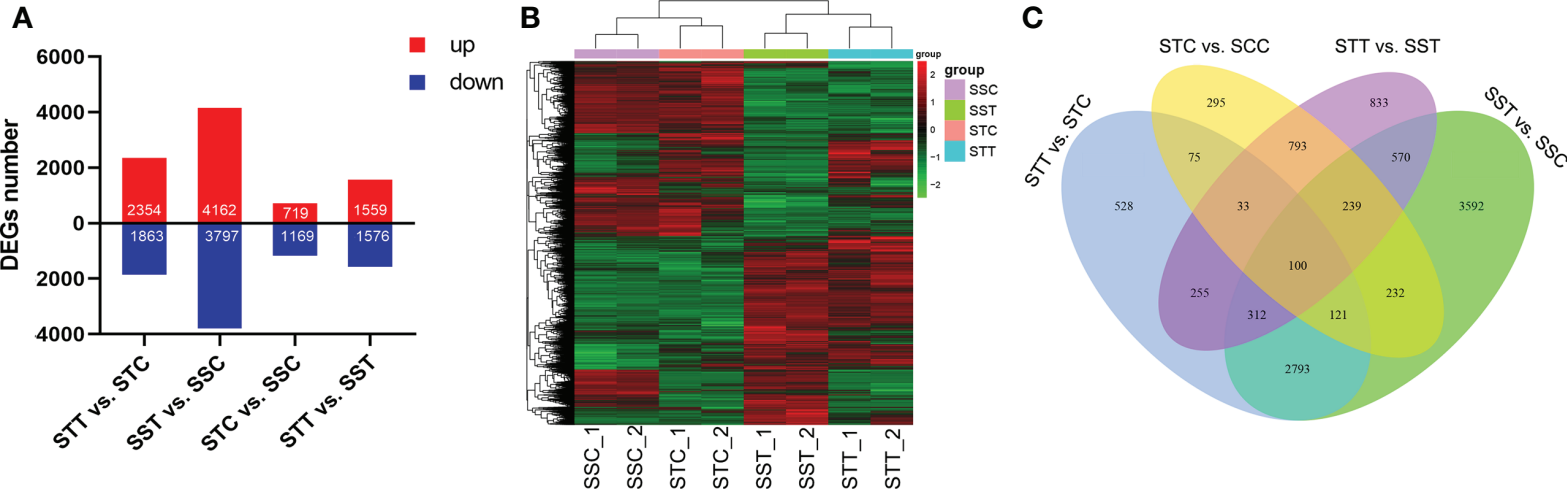
Summary of DEGs between salt treatment and control. **(A)** Number of DEGs in different comparisons. STC: LRNK1-Control, STT: LRNK1-Salt treatment, SSC: LR2381-Control, SST: LR2381-Salt treatment; **(B)** Expression heatmap of DEGs in different groups. The redder the color, the higher the expression; the greener the color, the lower the expression. **(C)** Venn diagram analysis results among different comparisons.

A total of 10,771 DEGs were identified by pairwise comparison of all the groups. There were 2,354, 4,162, 719, and 1,559 upregulated DEGs and 1,863, 3,797, 1,169, and 1,576 downregulated DEGs found during the comparison of STT vs. STC, SST vs. SSC, STC vs. SSC, and STT vs. SST, respectively (**Figure 2A**). The expression pattern of all DEGs showed a significant distinction between different groups (**Figure 2B**). Venn diagram analysis results showed that there were 3,326 common DEGs between STT vs. STC and SST vs. SSC (**Figure 2C**).

### Functional analysis of DEGs

**Figure 3:**
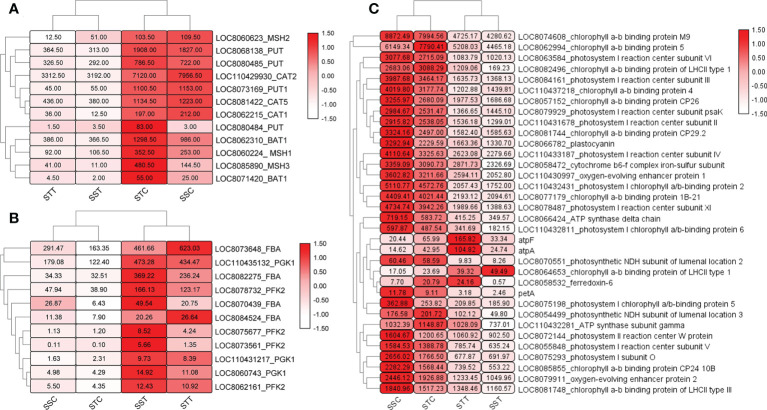
Heatmap of differently expressed genes (DEGs) in candidate pathways. The redder and larger the circle, the higher the expression. **(A)** Expression of DEGs in carbohydrate catabolic process. **(B)** Expression pattern of transcripts of SbCAT, SbPUT, SbMSH, and SbBAT. **(C)** Expression of DEGs related to photosynthesis.

**Figure 4:**
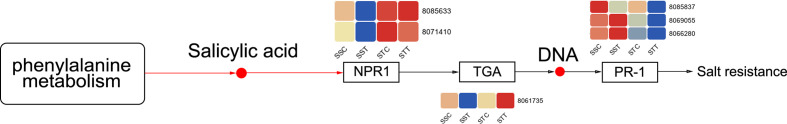
Differently expressed genes (DEGs) in the salicylic acid signal pathway. The redder the circle, the higher the expression. STC: LRNK1-Control, STT: LRNK1-Salt treatment, SSC: LR2381-Control, SST: LR2381-Salt treatment.

To reveal the role of DEGs, GO and KEGG enrichment analyses were performed. GO enrichment results revealed that 11 GO terms were significantly enriched in STT vs. STC, including carbohydrate catabolic process (GO:0016052) and transmembrane transport (GO:0055085). Most of the DEGs were upregulated in the carbohydrate catabolic process, including *SbFBA* (*fructose-bisphosphate aldolase*), *SbPGK* (*phosphoglycerate kinase*), and *SbPFK* (*pyrophosphate-fructose 6-phosphate*) family members (**Figure 3A**). In addition, some GO processes related to DNA replication were also related based on downregulated DEGs in STT vs. STC, including DNA metabolic process (GO:0006259), mismatch repair (GO:0006298), and mismatched DNA binding (GO:0030983). *SbCAT* (*catalase*), *SbPUT* (*polyamine transporter*), *SbMSH* (*DNA mismatch repair protein*), and *SbBAT* (*amino-acid permease*) were the key differentially expressed regulators in these GO terms (**Figure 3B**). For the salt-sensitive species *LR2381*, 16 GO terms were enriched, most of which were related to photosynthesis, including photosynthesis (GO:0015979), photosystem II (GO:0009523), and thylakoid (GO:0009579). DEGs like *SbpsbP* (*photosystem II oxygen-evolving complex protein*), *Sbpsa* (*photosystem I assembly factor*), and *SbLHCs* (*light-harvesting complex*) played key roles in these processes (**Figure 3C**). KEGG results showed that biosynthesis of amino acids (sbi01230), carbon fixation in photosynthetic organisms (sbi00710), glutathione metabolism (sbi00480), and circadian rhythm–plant (sbi04712) were significantly enriched based on the DEGs in STT vs. STC, and most DEGs involved were upregulated with salt treatment. Most of the genes in the C4-dicarboxylic acid cycle (belonging to sbi00710) were active. *SbPIF* (*ATP-dependent DNA helicase*), *SbHY5* (*transcription factor HY5*), and *SbFT* (*FT-interacting protein*) in circadian rhythm–plants were upregulated. For DEGs in the glutathione metabolism, *SbNADP^+^* was downregulated and *SbGST* (*probable glutathione S-transferase*) was upregulated. As for SST vs. SSC, the upregulated DEGs were enriched in carbon fixation in photosynthetic organisms (sbi00710), plant hormone signal transduction (sbi04075), MAPK signaling pathway–plant (sbi04016), glutathione metabolism (sbi00480), and carbon metabolism (sbi01200). The downregulated DEGs were enriched in porphyrin and chlorophyll metabolism (sbi00860), photosynthesis (sbi00195), carbon metabolism (sbi01200), starch and sucrose metabolism (sbi00500), and the TCA cycle (sbi00020). DEGs in plant hormone signal transduction showed that *SbPP2C* (*probable protein phosphatase 2C*), *SbABF*, *SbIAA* (*Auxin*), *SbEIN3* (*EIN3-binding F-box protein*), and all genes related to salicylic acid (including *SbJAR1*, *SbNPR1*, and *SbTGA*) increased by salt treatment (**Figure 4**). Also, *SbMAP3K* responded positively to salt stress. Photosynthesis-related genes, including *SbPsa*, *SbPsb*, and *SbPet*, were downregulated by salt stress.

### Summary of the LC-MS/MS metabolomics

The MS raw data (total ion current (TIC)) showed that there were 548 and 824 metabolites detected from all samples and quality controls under the negative and positive modes, respectively. PCA results showed that metabolisms differed between groups (**Supplementary Figures S2A, B**). XComparison analysis determined that there were 63 and 66 differential metabolites in STT vs. STC under negative and positive ion modes, respectively (**Supplementary Figures S2D, E**). As for SST vs. SSC, there were 40 and 46 differential metabolites under negative and positive ion modes, respectively (**Supplementary Figure S2C, D**).

### Differential metabolites responding to salt stress

**Figure 5:**
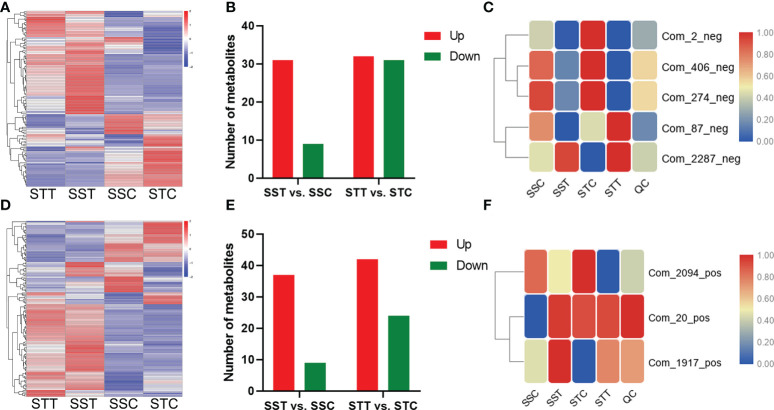
Differential metabolites respond to salt stress. **(A, D)** mean the differential metabolite content in different groups under negative and positive modes, respectively. The redder the circle, the higher the content. **(B, E)** showed the differential metabolite content in different comparisons under negative and positive modes, respectively. **(C, F)** showed the candidate metabolite profile content in different groups under negative and positive modes, respectively. The redder the circle, the higher the content.

In the negative ion mode, distinct profiles of these metabolites are shown by a heatmap in these four groups (**Figure 5A**). In salt-tolerant *LRNK1*, 32 and 31 metabolites were up- and downregulated, respectively, after salt treatment. As for the salt-sensitive *LR2381*, salt stress mediated 31 upregulated and nine downregulated metabolites (**Figure 5B**). The results of the KEGG enrichment analysis showed that plant hormone signal transduction (sbi04075) was significantly enriched, which was partially regulated by salicylic acid (Com_87_neg). Aside from the two metabolites, salt stress decreased the contents of 2-oxobutyric acid (Com_274_neg), which was involved in multiple pathways but was different in the two sorghum species. In addition, phenylalanine metabolism (map00360) and starch and sucrose metabolism (map00500) were active for energy homeostasis to cope with salt stress. Of these, five metabolites showed different profiles in STT vs. STC and SST vs. SSC (**Table 2**; **Figure 5C**), which is important.

**Table 2:** Five candidate metabolites in negative ion mode for salt-tolerant (ST) sorghum genotype under salt treatments (T).

ID	Name	Formula	Kegg_map
Com_406_neg	Uridine	C_9_H_12_N_2_O_6_	Pyrimidine metabolism/metabolic pathways
Com_2_neg	Benzoic acid	C_7_H_6_O_2_	Starch and sucrose metabolism/metabolic pathways
Com_2287_neg	Trehalose 6-phosphate	C_12_H_23_O_14_P	Glycine, serine, and threonine metabolism/cysteine and methionine metabolism/valine, leucine, and isoleucine biosynthesis/propanoate metabolism/C5-branched dibasic acid metabolism/metabolic pathways/biosynthesis of secondary metabolites/2-oxocarboxylic acid metabolism/biosynthesis of amino acids
Com_87_neg	Salicylic acid	C_7_H_6_O_3_	Phenylalanine metabolism/metabolic pathways/biosynthesis of secondary metabolites/plant hormone signal transduction
Com_274_neg	2-Oxobutyric acid	C_4_H_6_O_3_	Phenylalanine metabolism/metabolic pathways/biosynthesis of secondary metabolites

In the positive ion mode, distinct metabolic profiling was found after salt treatment (**Figure 5D**). In LRNK1, 42 and 24 metabolites were up- and downregulated, respectively. In *LR2381*, salt stress mediated 37 upregulated and nine downregulated metabolites (**Figure 5E**). Metabolic profiling in salt-tolerant *LRNK1*, glutathione metabolism (map00480), and flavonoid biosynthesis (map00941) were significantly enriched in KEGG analysis, in which glutathione disulfide (Com_2094_pos) and gallocatechin (Com_1917_pos) act as the key regulators, respectively. As for the salt-sensitive *LR2381*, Betaine (Com_20_pos) in glycine, serine, and threonine metabolism and ABC transporters pathway were upregulated by salt stress. The expression of these three differential metabolites in different groups indicated that they were the key metabolites affected by salt stress (**Table 3**; **Figure 5F**).

**Table 3:** Three candidate metabolites in positive ion mode for salt-tolerant (ST) sorghum genotypes under salt treatments (T).

ID	Name	Formula	Kegg_map
Com_2094_pos	Glutathione disulfide	cpd:C00127	Glutathione disulfide/GSSG/oxiglutatione/oxidized glutathione
Com_20_pos	Betaine	cpd:C00719	Betaine/trimethylaminoacetate/glycine betaine/*N*,*N*,*N*-trimethylglycine/**t**rimethylammonioacetate
Com_1917_pos	(+)-Gallocatechin	cpd:C12127	(+)-Gallocatechin/gallocatechol

### Conjoint analysis of metabolomic and transcriptomic data

**Figure 6:**
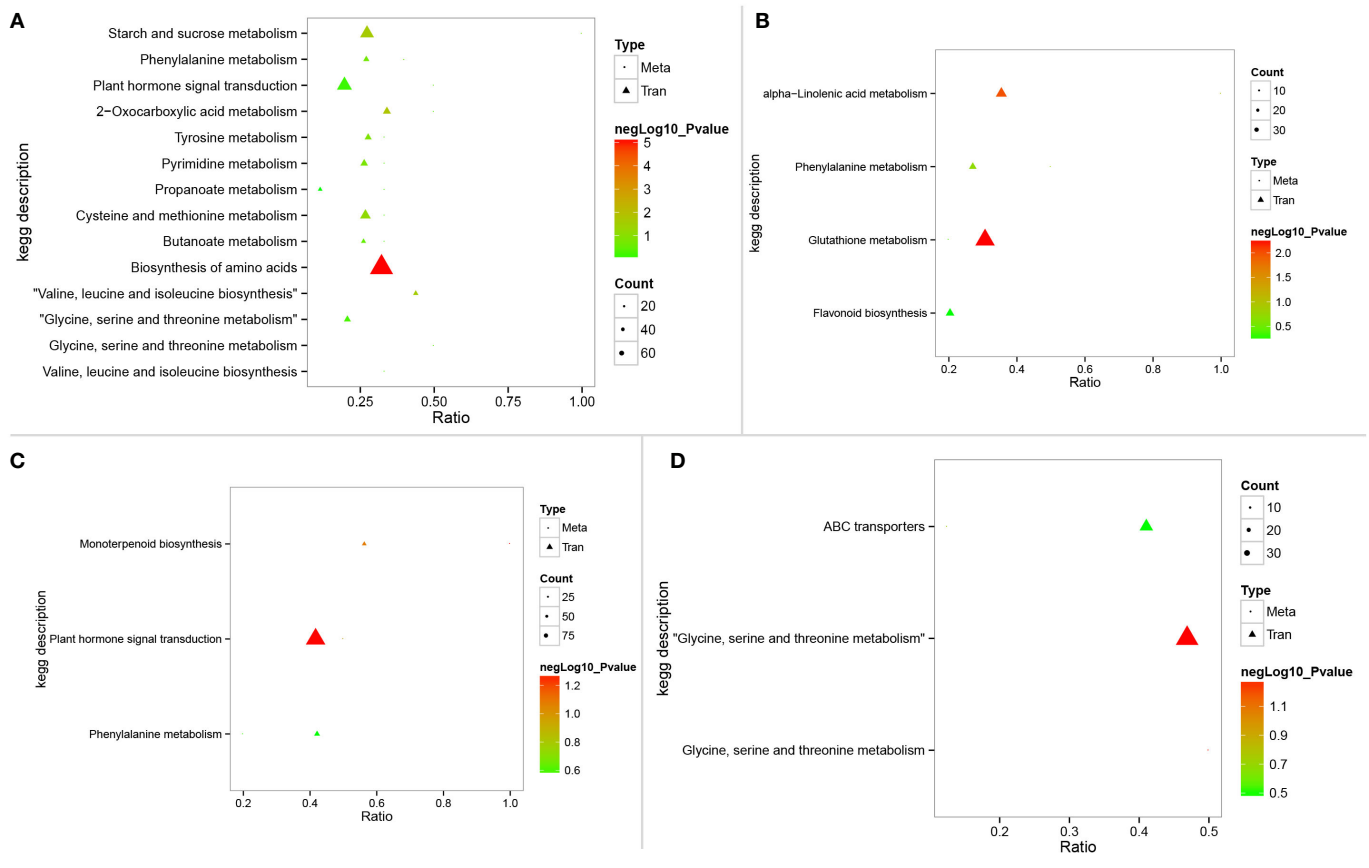
KEGG pathways enriched in integration analysis of metabolomics and transcriptomic data. **(A–D)** represent the KEGG enriched results in SST vs. SSC, STC vs. SSC, STT vs. SST, and STT vs. STC, respectively. STC: LRNK1-Control, STT: LRNK1-Salt treatment, SSC: LR2381-Control, SST: LR2381-Salt treatment.

To better understand the relationship between DEGs and differential metabolites, integration analysis of metabolomic and transcriptomic data was performed. Association results of the KEGG pathway showed that plant hormone signal transduction (sbi04075) was active in both *LRNK1* and *LR2381*. Glutathione metabolism (sbi00480) was active in salt-tolerant *LRNK1* but not enriched in *LR2381* (**Figures 6A–D**). This demonstrates that plant hormone signal transduction (sbi04075) and glutathione metabolism (sbi00480) are the key pathways responding to salt stress.

## Discussion

As sessile organisms, plants have developed sophisticated strategies to respond to diverse environmental stresses. Stress signals are perceived by several receptors at the cell membrane level, followed by their transduction to multiple second messengers such as abscisic acid (ABA), hydrogen peroxide (H_2_O_2_), and nitric oxide (NO) (Shi et al., 2014). In this study, the protective role of metabolites and gene expression patterns on the response to salt stress was revealed. A decrease in dry and fresh weights indicated that the leaves and roots of sorghum were damaged by short-term salt treatment. Relative water content is considered one of the easiest agricultural parameters that can be used to screen plants for drought and stress tolerance (Boutraa et al., 2010). The significant decrease in relative water content after salt stress in our results aligns with this view. Similar to our results, Nxele found that salinity stress could reduce water retention in sorghum plants (Nxele et al., 2017), which is a common response to salt stress. Under adverse circumstances, the chlorophyll level in the plant is a good indicator of photosynthetic function (Xu et al., 2008; Dourado et al., 2022). It has been found that the chlorophyll level of plants decreases with aggravated salt stress (Pires et al., 2015; Rachoski et al., 2015; Dąbrowski et al., 2016). In this study, we demonstrated that the chlorophyll content in the leaves of sorghum plants was significantly decreased by salt stress, which could be affected by oxygen species (ROS) such as O^2−^ and H_2_O_2_ that leads to lipid peroxidation and chlorophyll destruction (Foreman et al., 2003; Rustioni et al., 2015; Dąbrowski et al., 2016). The expression of DEGs related to chlorophyll was also decreased by salt stress, which was consistent with the chlorophyll level of leaves.

The transcriptome analysis results revealed the DEGs and pathways that respond to salt stress. Two sorghum species showed differences in several biological processes. In this study, the carbohydrate catabolic process was active after salt treatment. Carbohydrates act as signaling molecules and play a role in adaptive mechanisms to stress (Hanson and Smeekens, 2009; Ramel et al., 2009). Energy provision was essential for plants to survive under abiotic stresses. To fight against abiotic stress, the upregulation of catabolism-related proteins such as phosphoglycerate kinase to activate energy-generating processes seems reasonable (Ma et al., 2017). We found that members of the *SbFBAs*, *SbPGKs*, and *SbPFKs* families increased under salt stress, indicating an active energy metabolism during salt stress. Most of the DEGs had higher expression in the salt-tolerant *LRNK1* than in the salt-sensitive *LR2381* in the control group. Under salt treatment, the expression of these genes dramatically increased in both species. The salt tolerance of sorghum plants could be related to the energy supply of carbohydrates under normal conditions.

To defend against salt stress, plant hormone signal transduction (sbi04075) was active, where the salicylic acid signal pathway showed different response patterns between the two sorghum species. As expected, the metabolite salicylic acid (Com_87_neg) showed a great difference between *LRNK1* and *LR2381*, highlighting the critical role of the salicylic acid signal pathway during salt stress. Salicylic acid is a signaling molecule known to participate in defense responses against a variety of environmental stresses (Jayakannan et al., 2015a). Previous studies have reported that salicylic acid is an important signaling phytohormone that can increase salt tolerance in plants (Borsani et al., 2001; Horváth et al., 2007; Jayakannan et al., 2015b).

Salicylic acid-mediated resistance is related to the expression of pathogenesis-related (PR) genes (Vicente and Plasencia, 2011), which is partially controlled by redox-regulated nonexpresser of pathogenesis-related 1 (NPR1) protein, a positive regulator of salicylic acid signaling acting downstream of salicylic acid (Mou et al., 2003). *NPR1* has also been shown to serve as a salicylic acid receptor (Wu et al., 2012). The overexpression of *NPR1* in many species enhances the resistance of transgenic plants to stress (Chern et al., 2001; Fitzgerald et al., 2004). In our results, transcripts of *SbNPR1* (8085633 and 8071410) were more highly expressed in *LRNK1* than in *LR2381*, and the contents of salicylic acid increased in *LRNK1* and decreased in *LR2381* after salt stress. This could lead to higher resistance in *LRNK1* sorghum.

Applying certain concentrations of salicylic acid can reduce the salt stress of many plants, including peanuts and wheat (Mahmood et al., 2010). Salicylic acid can increase the content or activity of antioxidant enzymes in plants during salt tolerance, and maintain the stability of the membrane system in plants to improve their salt tolerance of plants. The contents of hydrogen peroxide (H_2_O_2_) and superoxide anion radical (O^2−^) and the harmful oxidation of lipids in plant cells could be appraised by increased malonaldehyde (MDA) content (Zhang X., 1992).

Increasing evidence has demonstrated that jasmonic acid functions in the regulation of plant responses to abiotic stresses (Qiu et al., 2014; Ding et al., 2015; Salimi et al., 2016; Aboagla et al., 2022). Although the results of metabolomics did not reflect differences in jasmonic acid between different species, transcripts of *SbJAZ* had higher expression in *LRNK1* than in *LR2381*. JAZ proteins are involved in abiotic stress tolerance mechanisms (Ye et al., 2009; Kazan, 2015; Chini et al., 2017). Enhanced stress tolerance of transgenic lines overexpressing JAZ proteins has been described in rice, cotton, and wild soybean (Ye et al., 2009; Wu et al., 2014; Zhao et al., 2016; Zhu et al., 2012). From the results, we speculated that the higher expression of *SbJAZ* promoted resistance to salt stress in *LRNK1* sorghum. The integration of metabolomic and transcriptomic results also supports this inference.

Betaine can protect plants from the adverse effects of abiotic stresses by ROS detoxification, adjusting cellular osmotica, and protecting membrane integrity (Ashraf and Foolad, 2007; Hayat et al., 2012). The overaccumulation of betaine can improve the stress resistance of wheat in a high-salt environment (Tian et al., 2016). In this study, the content of betaine (Com_20_pos) in STC (*LRNK1* without salt treatment) was significantly higher than that of SSC (*LR2381* without salt treatment). After salt treatment, the betaine in *LR2381* increased; however, due to the lack of betaine reserves before salt treatment, the *LR2381* plants suffered considerable damage under salt stress. Sufficient betaine reserves can likely help sorghum plants resist salt stress.

## Conclusion

The damage of salt stress to sorghum was similar to that of other plants, including decreases in relative water content, chlorophyll, and leaf weight. The dynamic gene expression pattern responded to salt stress. As a salt-tolerant species, *LRNK1* sorghum can defend against salt stress by a variety of biological processes. According to our results, sufficient carbohydrate energy reserves under normal conditions are important for resisting salt stress, and the expression of *SbFBAs*, *SbPGKs*, and *SbPFKs* could reflect the activity of the carbohydrate catabolic process. Importantly, the salt tolerance of sorghum was mediated by salicylic acid. Plants with higher salt tolerance have a higher salicylic acid content. Additionally, the expression of *SbNPR1*, a salicylic acid receptor, was positively correlated with salt tolerance. Similarly, betaine had been shown to respond to salt stress in sorghum, the accumulation of which under normal conditions was important for resisting salt stress. Moreover, a higher expression of *SbJAZ* promoted resistance to salt stress in *LRNK1* sorghum. The results of this research will play an important role in revealing the metabolic regulatory mechanism of sorghum salt tolerance and will contribute to sorghum breeding for salt tolerance, the efficient production of salt tolerance, and the development and utilization of saline land.

## Data availability statement

The datasets presented in this study can be found in online repositories. The names of the repository/repositories and accession number(s) can be found below: NCBI [accession: PRJNA810939].

## Author contributions

Lab work primarily conducted by FZ at Liaoning Agriculture Science as a PhD. FL, YW, ZZ, JW, KuZ, HW, JZ, YD, FK provided help for the manuscript preparation. KaZ provided technical guidance and advice for experiment design during the research. All authors contributed to the article and approved the submitted version.

## Funding

This research was funded by the following projects: National Key R&D Program of China (2019YFD1001700), China Agriculture Research System of MOF and MARA (CARS-06), Liaoning Provincial Natural Science Foundation Project, China (2019-MS-197), Science and Technology Plan of Shenyang in 2022-Research on Sorghum Germplasm Innovation and breeding of high-quality special varieties, General Project of the Dean’s Fund of Liaoning Academy of Agricultural Sciences, China (2021MS0504), and Agricultural Public Relations and Industrialization Project in Liaoning Province, China (2020JH2/10200014).

## Conflict of interest

The authors declare that the research was conducted in the absence of any commercial or financial relationships that could be construed as a potential conflict of interest.

## Publisher’s note

All claims expressed in this article are solely those of the authors and do not necessarily represent those of their affiliated organizations, or those of the publisher, the editors and the reviewers. Any product that may be evaluated in this article, or claim that may be made by its manufacturer, is not guaranteed or endorsed by the publisher.

## Supplementary material

Supplementary Figure 1 Samples correlation and PCA results based on the sequence data. **(A)** Pearson correlation between samples. **(B)** PCA results based on the sequence data.

Supplementary Figure 2 KEGG pathways enriched in integration analysis of metabolomics and transcriptomic data. **(A–D)** represent the KEGG enriched results in SST vs. SSC, STC vs. SSC, STT vs. SST, and STT vs. STC, respectively. STC: LRNK1-Control, STT: LRNK1-Salt treatment, SSC: LR2381-Control, SST: LR2381-Salt treatment.
