# Supplementary material for: Combined transcriptomic and physiological metabolomic analyses elucidate key biological pathways in the response of two sorghum genotypes to salinity stress
Source: Front Plant Sci. 2022 Oct 13;13:880373. doi: 10.3389/fpls.2022.880373 (PMC9608512; doi:10.3389/fpls.2022.880373)
Supplement: Supplementary file 1 [file DataSheet_1.docx]

**Table S1 Recipe of sorghum hydroponic culture (Kimura solution) for salt-tolerant (ST) genotype sorghum under salt treatments(T) at the seeding period**

| Nutritious mother liquor | Components | MW | TM | g/L | Dilution multiple of working liquid | Working solution concentration (mM) |
| --- | --- | --- | --- | --- | --- | --- |
| Stock-1 | MgSO_4_🞄7H_2_O | 246.3 | 0.547 | 134.800 | 1000× | 0.5470 |
|  | (NH_4_)_2_SO_4_ | 132.0 | 0.365 | 48.200 |  | 0.3650 |
| Stock-2 | KH_2_PO_4_ | 136.1 | 0.182 | 24.800 | 1000× | 0.1820 |
| Stock-3 | KNO_3_ | 101.1 | 0.183 | 18.500 | 1000× | 0.1830 |
|  | Ca(NO_3_)_2_🞄4H_2_O | 236.0 | 0.366 | 86.400 |  | 0.3660 |
| Stock-4 | MnCl_2_.4H_2_O | 197.9 | 0.005 | 0.990 | 10000× | 0.0005 |
|  | H_3_BO_3_ | 61.8 | 0.03 | 1.860 |  | 0.0030 |
|  | (NH_4_)_6_Mo_7_O_24_🞄4H_2_O | 1235.9 | 0.001 | 1.236 |  | 0.0001 |
|  | ZnSO_4_🞄7H_2_O | 287.5 | 0.004 | 1.150 |  | 0.0004 |
|  | CuSO_4_🞄5H_2_O | 249.5 | 0.002 | 0.518 |  | 0.0002 |
| Stock-5 | NaFe-EDTA🞄3H_2_O | 421.1 | 0.100 | 42.100 | 2500× | 0.0400 |
| MES | (pH5.5) | 195.2 | 0.500 | 97.620 | 250× | 2.0000 |

MW: Molecular weight

TM: Total moles

MES:2-(N-morpholino) ethanesulfonic acid

**Table S2 Illumina sequencing data summary** **of Salt Sensitivity (SS) and salt-tolerant (ST) genotype sorghum under control (C)and salt treatments(T)**

| **sample** | **raw_reads (Mb)** | **clean_reads (Mb)** | **clean_bases (Gb)** | **error_rate (%)** | **Q20 (%)** | **Q30 (%)** | **GC_pct (%)** | **total mapped reads** | **unique mapped reads** | **unique_map rate (%)** | **multi mapped reads** | **multi map rate (%)** |
| --- | --- | --- | --- | --- | --- | --- | --- | --- | --- | --- | --- | --- |
| STC_1 | 57.60043 | 55.6923 | 8.35 | 0.02 | 98 | 94.41 | 54.75 | 52199276 | 51268667 | 0.9206 | 930609 | 0.0167 |
| STC_2 | 53.942936 | 51.070232 | 7.66 | 0.02 | 97.99 | 94.43 | 54.68 | 47731844 | 46894770 | 0.9182 | 837074 | 0.0164 |
| SSC_1 | 56.698198 | 55.310032 | 8.3 | 0.02 | 98.04 | 94.54 | 55.57 | 52627624 | 51615585 | 0.9332 | 1012039 | 0.0183 |
| SSC_2 | 56.698772 | 55.153354 | 8.27 | 0.02 | 98.03 | 94.46 | 55.14 | 52366596 | 51395458 | 0.9319 | 971138 | 0.0176 |
| STT_1 | 54.159836 | 50.802786 | 7.62 | 0.03 | 97.89 | 94.23 | 54.38 | 47417399 | 46593680 | 0.9171 | 823719 | 0.0162 |
| STT_2 | 57.98724 | 57.167266 | 8.58 | 0.02 | 98.03 | 94.44 | 53.86 | 53829749 | 52829049 | 0.9241 | 1000700 | 0.0175 |
| SST_1 | 64.116328 | 63.006788 | 9.45 | 0.02 | 97.95 | 94.29 | 54.79 | 59875516 | 58779971 | 0.9329 | 1095545 | 0.0174 |
| SST_2 | 56.897586 | 55.821914 | 8.37 | 0.03 | 97.78 | 93.93 | 54.77 | 52854405 | 51856076 | 0.9290 | 998329 | 0.0179 |
