# Supplementary figures and images for: Combined transcriptomic and physiological metabolomic analyses elucidate key biological pathways in the response of two sorghum genotypes to salinity stress
Source: Front Plant Sci. 2022 Oct 13;13:880373. doi: 10.3389/fpls.2022.880373 (PMC9608512; doi:10.3389/fpls.2022.880373)

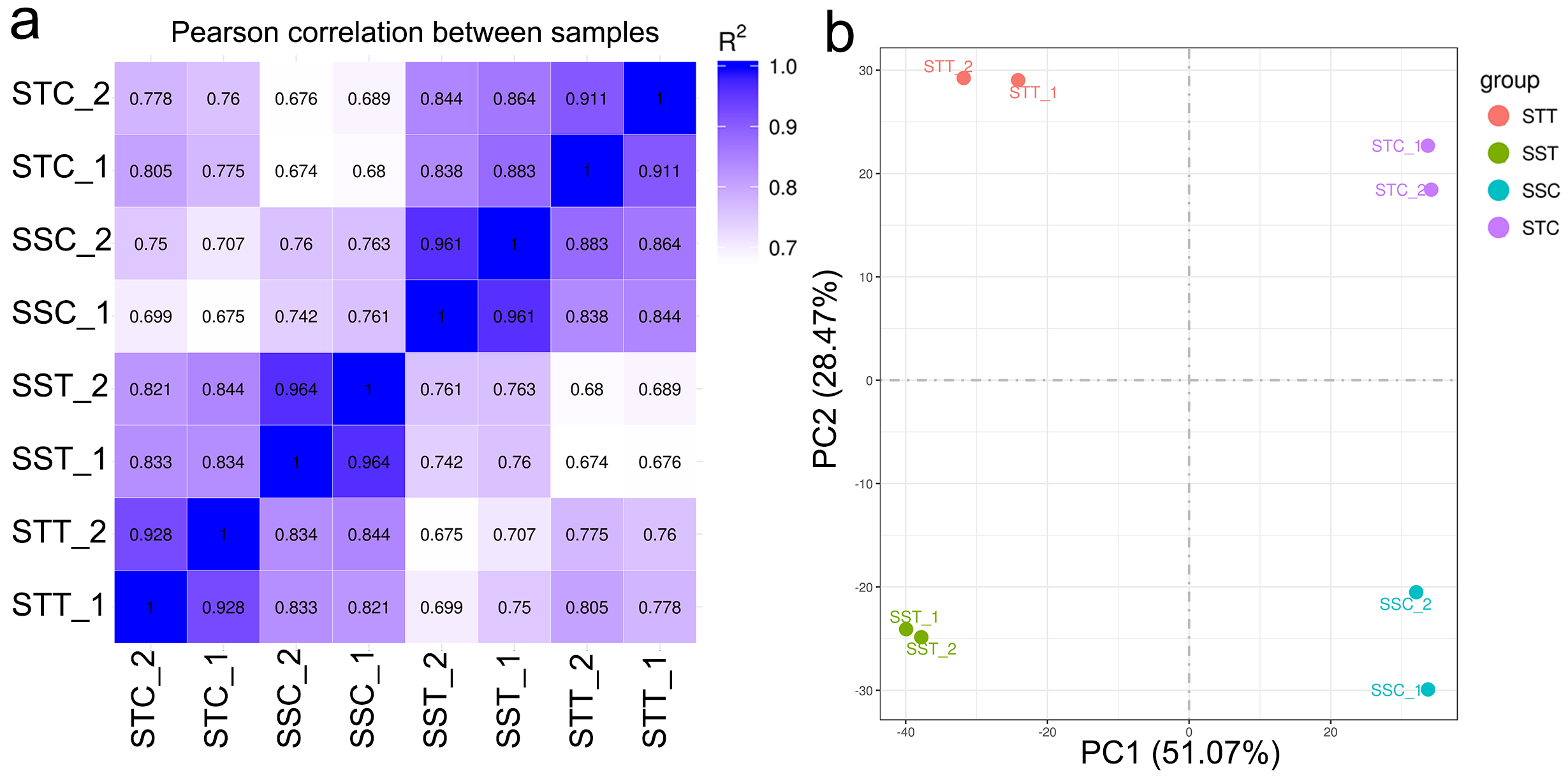

Supplement: Supplementary Figure 1 — Samples correlation and PCA results based on the sequence data. (A) Pearson correlation between samples. (B) PCA results based on the sequence data. [file Image_1.tif]

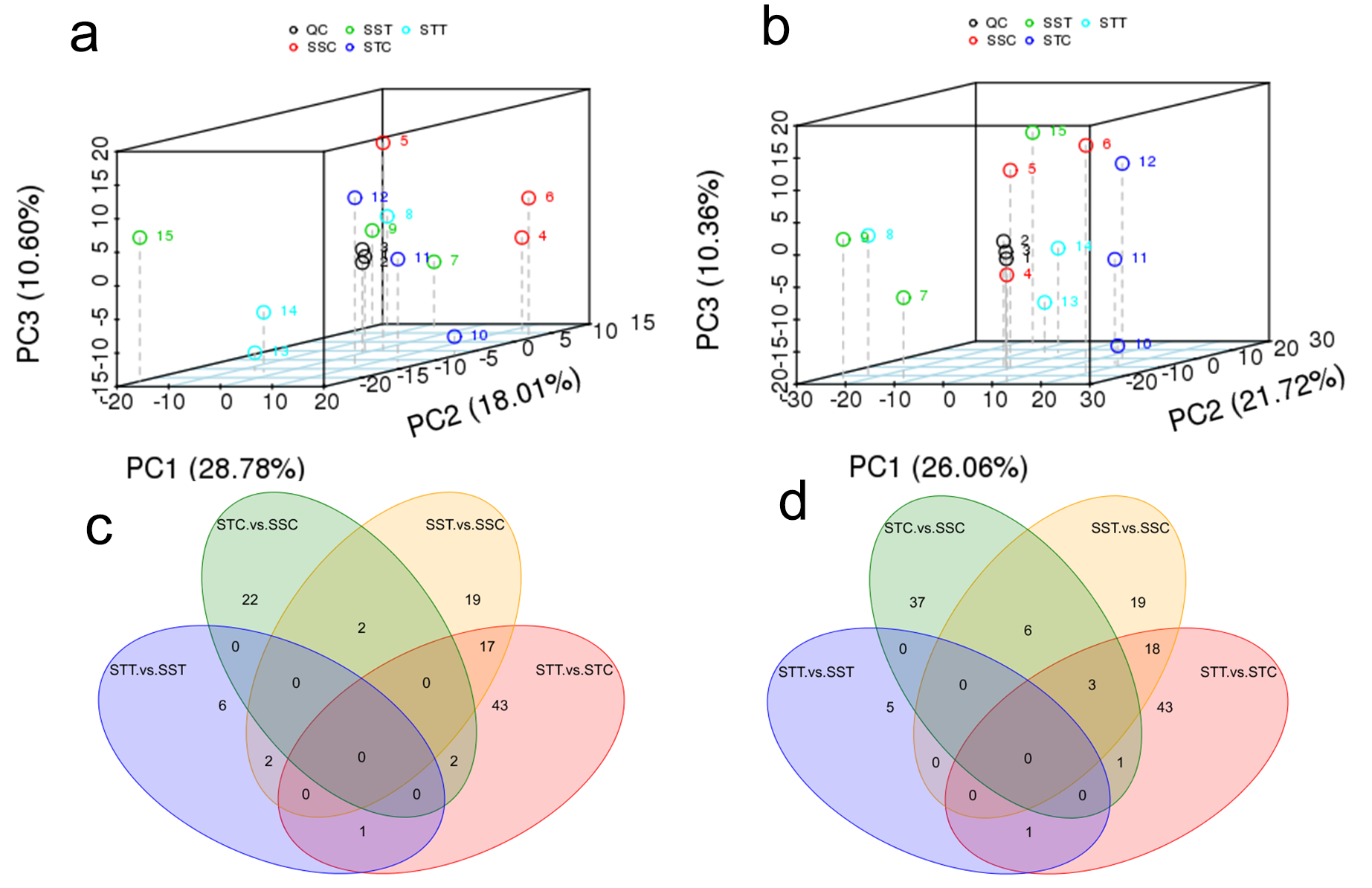

Supplement: Supplementary Figure 2 — KEGG pathways enriched in integration analysis of metabolomics and transcriptomic data. (A–D) represent the KEGG enriched results in SST vs. SSC, STC vs. SSC, STT vs. SST, and STT vs. STC, respectively. STC: LRNK1-Control, STT: LRNK1-Salt treatment, SSC: LR2381-Control, SST: LR2381-Salt treatment. [file Image_2.tif]
